# Simultaneous inhibition of ATR and PARP sensitizes colon cancer cell lines to irinotecan

**DOI:** 10.3389/fphar.2015.00147

**Published:** 2015-07-22

**Authors:** Atlal Abu-Sanad, Yunzhe Wang, Fatemeh Hasheminasab, Justin Panasci, Alycia Noë, Lorena Rosca, David Davidson, Lilian Amrein, Bahram Sharif-Askari, Raquel Aloyz, Lawrence Panasci

**Affiliations:** Montreal Centre for Experimental Therapeutics in Cancer, Segal Cancer Center, Lady Davis Institute, Jewish General Hospital, McGill UniversityMontréal, QC, Canada

**Keywords:** PARP, ATR, SN38, colon cancer, drug sensitization, irinotecan, DNA-PK, inhibitor

## Abstract

Enhanced DNA damage repair is one mechanism involved in colon cancer drug resistance. Thus, targeting molecular components of repair pathways with specific small molecule inhibitors may improve the efficacy of chemotherapy. ABT-888 and VE-821, inhibitors of poly-ADP-ribose-polymerase (PARP) and the serine/threonine-kinase Ataxia telangiectasia related (ATR), respectively, were used to treat colon cancer cell lines in combination with the topoisomerase-I inhibitor irinotecan (SN38). Our findings show that each of these DNA repair inhibitors utilized alone at nontoxic single agent concentrations resulted in sensitization to SN38 producing a 1.4–3 fold reduction in the 50% inhibitory concentration (IC_50_) of SN38 in three colon cancer cell lines. When combined together, nontoxic concentrations of ABT-888 and VE-821 produced a 4.5–27 fold reduction in the IC_50_ of SN38 with the HCT-116 colon cancer cells demonstrating the highest sensitization as compared to LoVo and HT-29 colon cancer cells. Furthermore, the combination of all three agents was associated with maximal G_2_ −M arrest and enhanced DNA-damage (γH2AX) in all three colon cancer cell lines. The mechanism of this enhanced sensitization was associated with: (a) maximal suppression of SN38 induced PARP activity in the presence of both inhibitors and (b) ABT-888 producing partial abrogation of the VE-821 enhancement of SN38 induced DNA-PK phosphorylation, resulting in more unrepaired DNA damage; these alterations were only present in the HCT-116 cells which have reduced levels of ATM. This novel combination of DNA repair inhibitors may be useful to enhance the activity of DNA damaging chemotherapies such as irinotecan and help produce sensitization to this drug in colon cancer.

## Introduction

DNA-interacting chemotherapy drugs remain the mainstay therapy for most advanced cancers, including metastatic colon cancer. The introduction of irinotecan and oxaliplatin has doubled survival of patients with metastatic colon cancer, but still the majority of patients with this disease die within 3 years of diagnosis of metastases (Goldberg et al., 2004). Reports by us and others demonstrating that DNA repair is an important factor in resistance to chemotherapy have lent support to the development of inhibitors of key enzymes involved in the DNA damage response (DDR) with promising preclinical results (Panasci et al., 2001; Aloyz et al., 2004; Willmore et al., 2004; Amrein et al., 2011; Siddiqui-Jain et al., 2012; Davidson et al., 2012a). At the apex of the DDR are three related protein kinases: Ataxia telangiectasia mutated (ATM), Ataxia-telangiectasia-and-Rad3-related (ATR) and DNA-dependent protein kinase (DNA-PK). These kinases belong to the phosphoinositide-3-kinase-related protein kinase family and share structural homologies and modes of regulation (Mordes and Cortez, 2008; Lempiainen and Halazonetis, 2009; Lovejoy and Cortez, 2009). Whereas, all three kinases are activated by DNA double-strand breaks (DSBs), ATR can also be activated by base adducts and cross-links in the DNA. Furthermore, Poly ADP ribose polymerase 1 (PARP1), which catalyzes the formation of poly ADP ribose chains (PAR) on cellular DNA and proteins, is rapidly recruited to and activated by DSBs and DNA nicks. The addition of PAR by PARP1 can alter the functions of modified proteins such as topoisomerase-I and DNA-PK. Inhibitors of DNA-PK or PARP individually can sensitize cancer cells to DNA-damaging agents (Willmore et al., 2004; Donawho et al., 2007; Penning et al., 2009; Amrein et al., 2011; Siddiqui-Jain et al., 2012; Davidson et al., 2012b). We have previously demonstrated increased sensitization of primary chronic lymphocytic leukemia cells to chlorambucil by the use of two DNA repair inhibitors employed together targeting HRR and/or DNA-PK (Amrein et al., 2011).

As cancer cells deficient in ATR or DSB repair are sensitive to inhibitors of PARP1 (Audeh et al., 2010; Tutt et al., 2010; Huehls et al., 2012), we hypothesized that inhibition of ATR and PARP1 would result in more sensitization to the DSB inducing agent, irinotecan. Noteworthy, irinotecan induces cell cycle arrest in an ATR-dependent manner through Chk1 phosphorylation, an accepted marker of ATR activity (Zhao and Piwnica-Worms, 2001; Nam and Cortez, 2011). Also, our reported results showing that an inhibitor of PARP1 (ABT-888) sensitizes colon cancer cell lines to irinotecan (Davidson et al., 2013) have lead to an ongoing clinical trial (clicaltrials.gov, Abbvie, NCT02305758). ATR is involved in many biological processes including DDR and replication stress. Inhibitors of ATR are in development (Fokas et al., 2014). One of these, VE-821 has resulted in sensitization of human cancer cell lines to many chemotherapies (Reaper et al., 2011). Additionally, it has been reported (Reaper et al., 2011; Ahmed et al., 2013) that the ATR inhibitor VE-822 improved the efficacy of gemcitabine and radiation in treatment of pancreatic cancer cells while not affecting normal cells both *in-vitro* and *in-vivo*. The reports of the potent sensitizing effect of ATR inhibitors, including VE-821, on chemotherapy (Reaper et al., 2011) prompted us to assess the effect of the combination of ABT-888 and VE-821, targeting PARP and ATR, respectively, on irinotecan cytotoxicity in colon cancer cells. For this purpose we utilized the micro-satellite-instability, p53, BRAF and PTEN wild-type HCT-116 and LoVo colon cancer cell lines plus the p53 mutated micro-satellite-stable, BRAF mutated, PTEN wild type HT-29 cell line (Ahmed et al., 2013).

## Materials and methods

### Cell lines, drug treatments, and reagents

The genetically diverse colon cancer cell lines HCT-116, HT-29, and LoVo were obtained from the American Type Culture Collection (ATCC) and maintained as monolayers at 37°C in 5% CO_2_ in RPMI, McCoy's, or Ham's F-12 medium, respectively, supplemented with 10% fetal bovine serum and penicillin/streptomycin. All experiments were performed on cells in exponential growth phase.

SN38 (the activated form of irinotecan), chemicals and reagents were obtained from Sigma-Aldrich or Invitrogen. Abbott Laboratories provided ABT-888, and VE-821 was generously donated by Vertex pharmaceutical (Abingdon, Oxfordshire, UK).

### Sulforhodamine (SRB) cytotoxicity assay

SRB assays were performed as described by us and by Vichai et al. (Vichai and Kirtikara, 2006; Davidson et al., 2012a). It has been previously shown that cytotoxicity results obtained with the SRB assay correlate well with both the MTT and clonogenic assays. Furthermore, this assay has been adopted by the NCI for large scale screening of new drugs (Perez et al., 1993). Cells were treated with SN38, the PARP inhibitor ABT-888, and the ATR inhibitor VE-821 alone or in combination (concentrations indicated in results section). Five days after drug treatment, the percentage of surviving cells was measured using a 96-well plate reader. Plating efficiency experiments were performed to determine the ideal plating density and ensure cells were growing exponentially at the 5 day time point. The 5-day time point was selected to permit approximately 4 growth cycles before fixing cells. The efficacy of the various drug treatments was determined by calculating the 50% inhibitory concentrations (IC_50_) and sensitization values. Sensitization values (*R*-values) were calculated using the equation described by Willmore et al. (2008). According to this equation, values greater than 1 indicate sensitization. Each experiment consisted of triplicate drug treatments, and experiments were repeated at least three times. Wells treated with DMSO (vehicle), ABT-888 alone, VE-821 alone or VE-821/ABT-888 (without SN38) were used as controls in all experiments.

### Cell cycle analysis

Cells were grown on 6-well tissue culture plates, incubated with each drug separately (concentrations indicated in figure legends) or with drug combinations for 24 h (exponential growth), trypsinized, fixed, and permeabilized with 75% ethanol in Ca^2+^/Mg^2+^ free PBS, and stored at −20°C. Cells were stained with 5 μg/mL 7-amino-actinomycin D (7AAD) or propidium iodide (PI), treated with 0.2 mg/mL RNAseA as per our laboratory protocol and examined by flow cytometry (Davidson et al., 2012a). A minimum of 20,000 events was recorded for each sample.

### Quantification of the double stranded DNA (dsDNA) damage marker γH2AX

Cells were prepared as described for cell cycle analysis, incubated with mouse anti-γH2AX antibodies at 4°C overnight, and washed with phosphate-buffered saline (PBS). Cells were then incubated with goat anti-mouse Alexa 488-conjugated secondary antibodies for 1 h and washed in PBS. The mean fluorescence intensity was measured to determine levels of γH2AX at 24 h post-treatment as described (Davidson et al., 2013).

### Western blotting

HCT-116 cells were grown in T25 flasks for 24 h (exponential growth), treated with anti cancer drugs (as indicated in figure legends) and harvested by scraping 24 h post treatment. Cells were centrifuged at 1500 rpm for 5 min and pellets washed in cold PBS. Pellets were dissolved in lysis buffer as previously described (Davidson et al., 2012b, 2013). Cell lysates were separated by SDS-PAGE, transferred to nitrocellulose membranes under the appropriate conditions, and blotted for the following antigens: total Chk1 (Santa Cruz, sc-8408), DNA-PK (Upstate, 05-423) and Rad51 (Upstate), phosphorylated antibodies; Chk1 (S317) (Cell Signaling, 2344), DNA-PK (S2056) (Abcam, ab18192) and Rad51 (T309) (Abcam) and β-actin (Santa Cruz, sc-1616). Chk1, DNA-PK and Rad51 levels were normalized to β-actin and phosphorylated-Chk1, -DNA-PK, and -Rad51 were normalized to total-Chk1, total-DNA-PK and total-Rad51 respectively. Each experiment was repeated at least 3 times. Blots were quantified using ImageJ image analysis software.

### PARP inhibition assay

T25 flasks were inoculated with 5×10^5^ HCT-116 cells and grown for 24 h as previously described (Davidson et al., 2013). Cells were treated with the PARP inhibitor ABT-888, VE-821, or SN38 alone or in combination for 24 h (concentrations indicated in figure legend). Cells were harvested by scraping, centrifuging at 1500 rpm for 5 min and lysed in 25 μL of lysis buffer. The resulting supernatant was assayed for PARP activity using the Trevigen Inc. universal PARP assay as previously done (Davidson et al., 2013). All activity measurements were normalized to total PARP protein levels as determined by western blot analysis.

### Statistical analysis

Data analysis was performed using GraphPad Prism (GraphPad Software, San Diego, CA, USA). Statistically significant changes were determined using two-tailed unpaired Student's *t*-tests.

## Results

### The combination of non-cytotoxic concentrations of VE-821 plus ABT-888 had a greater effect on Sn38 cytotoxicity than either inhibitor alone

**Figure 1:**
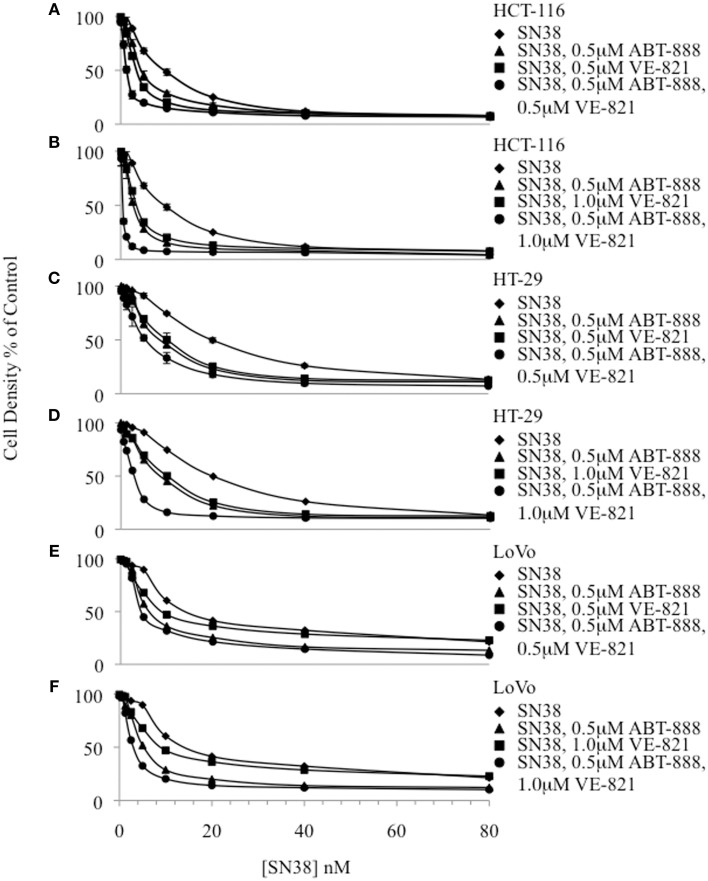
**Cytotoxicity curves plotting cell density of drug treated samples relative to the vehicle-treated control for 3 colon cancer cell lines**. **(A)** HCT-116 combinations including 0.5 μM VE-821, **(B)** HCT-116 combinations including 1.0 μM VE-821, **(C)** HT-29 combinations including 0.5 μM VE-821, **(D)** HT-29 combinations including 1.0 μM VE-821, **(E)** LoVo combinations including 0.5 μM VE-821, and **(F)** LoVo combinations including 1 μM VE-821.

The IC_50_ of SN38 alone was measured separately in the colon cancer cell lines HCT-116, HT-29 and LoVo (Figure 1, Supplemental Table 1). The HCT-116 cell line was the most sensitive to SN38 while the HT-29 cell line was the most resistant. Additionally, ABT-888 and VE-821 were tested individually in each cell line to obtain the corresponding IC_50_ values (Supplemental Table 2). Again, the HCT-116 cells were the most sensitive to ABT-888 and HT-29 cells were the most resistant to ABT-888 (38–100 μM). Moreover, HT-29 cells were 10 fold more resistant and LoVo cells were 6 fold more resistant to VE-821 as compared to HCT-116 cells (10.5–100 μM). Based on the IC_50_ values obtained for ABT-888 and VE-821 non-toxic concentrations of these compounds were selected for combination drug treatments with SN38. At the selected concentrations [VE-821 alone (0.5 or 1 μM), ABT-888 alone (0.5 μM), and VE-821 (0.5 or 1 μM) plus ABT-888 (0.5 μM)], there was no evidence of decreased cell growth and/or increased death. Our previous study demonstrated that 0.5 μM ABT-888 optimally sensitized colon cancer cell lines to SN38 (Davidson et al., 2013). Combining 0.5 μM ABT-888 with SN38 reduced the SN38 IC_50_ in all tested cell lines with the greatest sensitization observed in the HCT-116 cell line (1.4–2.1 fold sensitization). Furthermore, the IC_50_ of SN38 was reduced significantly by the addition of VE-821 at 0.5 or 1 μM in all cell lines (1.6–3 fold sensitization). The observed effect was dose-dependent with the higher concentration of VE-821 modestly decreasing the IC_50_ of SN38 (Figure 1, Supplemental Table 1). The combination of both inhibitors at non-toxic concentrations resulted in the greatest decrease in SN38 IC_50_ values (HCT-116: 27 fold, LoVo and HT-29: 4.5 and 5.3 fold) (Figure 1, Supplemental Table 1).

### The sensitization effect of VE-821 plus ABT-888 on SN38 cytotoxicity was associated with promotion of G_2_-M cell cycle arrest and increased γH2AX levels

**Figure 2:**
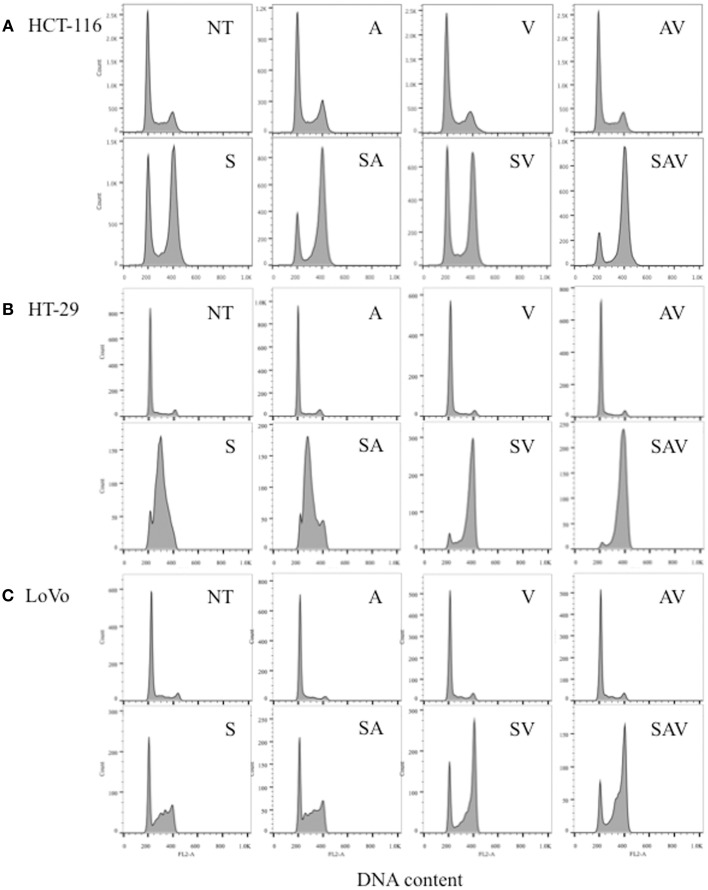
**Cell cycle analysis of (A) HCT-116, (B) HT-29, and (C) LoVo cell lines 24 h post treatment with IC_50_ concentrations of SN38 (S) and/or 0.5 μM ABT-888 (A) 1 μM VE-821 (V) or both (AV)**. (NT) cells treated with vehicle alone, (AV) ABT-888 and VE-821, (SA) SN38 and ABT-888, (SV) SN38 and VE-821, and (SAV) SN38, ABT-888 and VE-821 in combination.

HCT-116, HT-29, and LoVo cells treated with SN38 for 24 h accumulated in the S and G_2_-M phases of the cell cycle (Figures 2A–C). In HCT-116 and HT-29 cells, the combination treatment with ABT-888 plus VE-821 and SN38 had the greatest increase in G_2_-M arrest as compared with SN38 alone (Figures 2A,B). In LoVo cells there was less effect on G2-M arrest (Figure 2C). These results are consistent with our previous publication on ABT-888 with irinotecan and the results of Reaper et al., showing the effects of VE-821 plus chemotherapy (Davidson et al., 2013; Reaper et al., 2011).

**Figure 3:**
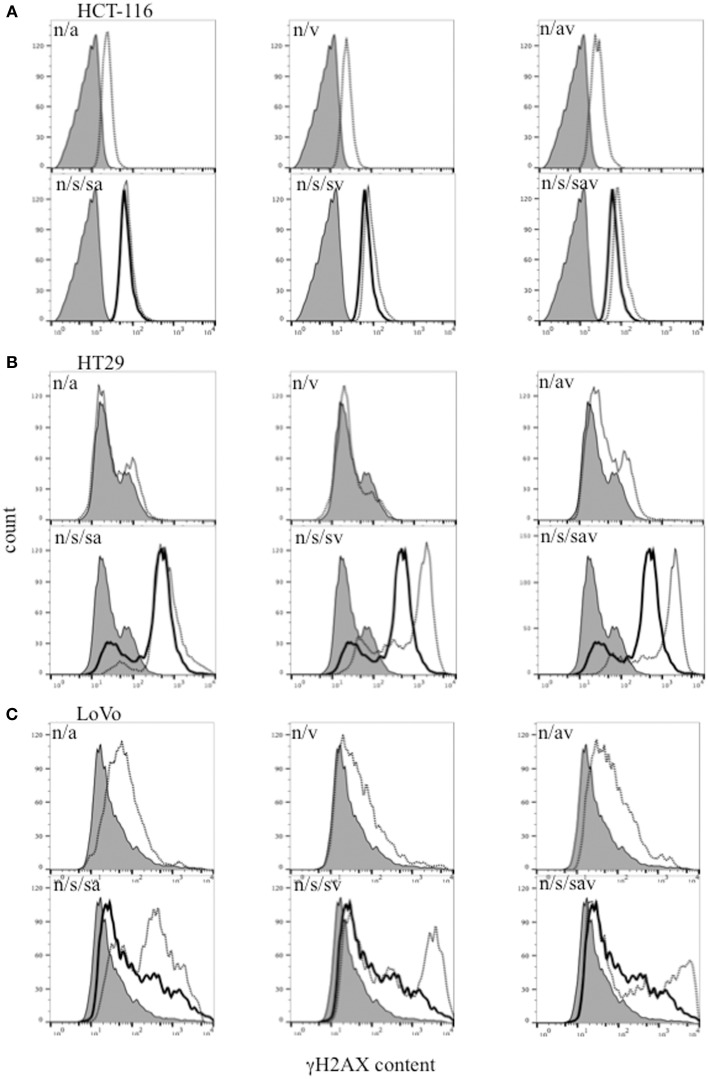
**Flow cytometric analysis of γH2AX in (A) HCT-116, (B) HT29, and (C) LoVo colon cancer cells 24 h-post treatment with SN38 at IC_50_ concentrations (s) (solid black line) and/or 0.5 μM ABT-888 (a) and/or 1 μM VE-821 (v) drug combinations [dashed black lines. n = cells treated with vehicle alone (solid gray area)]**.

Histone H2AX is phosphorylated on Ser139 (γH2AX) in the presence of DSBs, and is therefore used as a surrogate marker of DSBs (Paull et al., 2000). Our data show that treatment with the combination ABT888/VE-821, SN38 alone or the different combinations of these inhibitors with SN38, increased γH2AX levels in the 3 colon cancer cell lines. The highest level of γH2AX was observed in the SN38/ABT-888/VE-821 combination-treated cells (Figure 3).

### ABT-888 abrogated basal and drug-induced PARP activity in colon cancer cells

**Figure 4:**
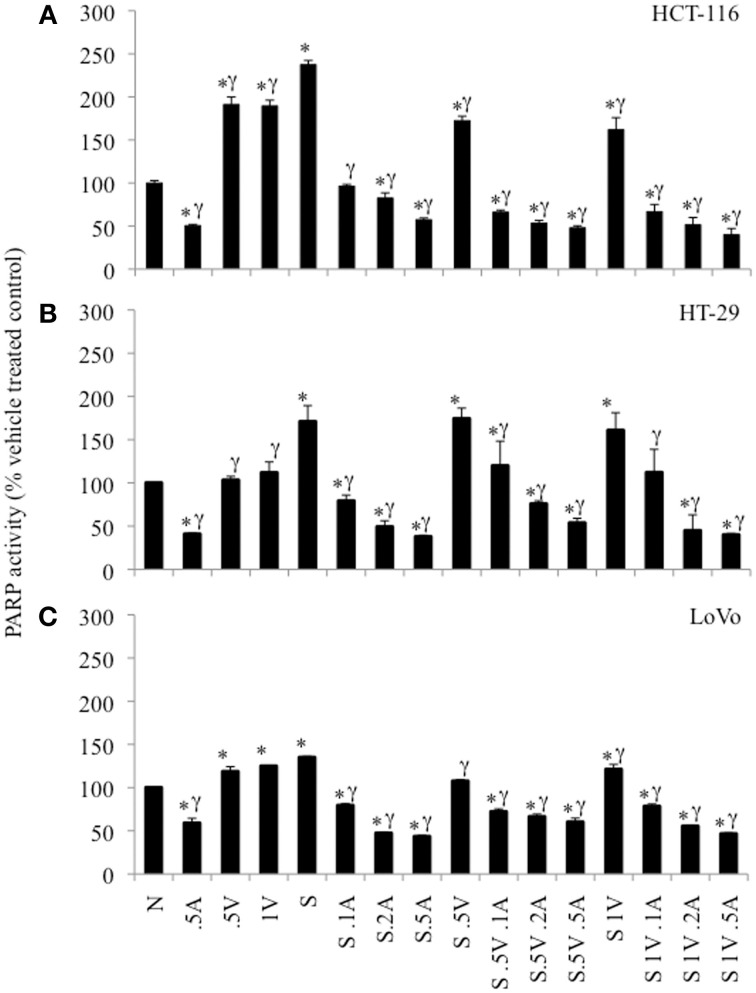
**PARP-activity in colon cancer cell lines as percent of untreated control cells**. **(A)** HCT-116 cells, **(B)** HT-29 cells, and **(C)** LoVo cells. Cells treated with vehicle alone (N), 0.5 μM ABT-888 (0.5A), 0.5 μM VE-821 (0.5V) or 1 μM VE-821 (1V), 64 nM SN38 (S) alone or in combination with varying concentrations of ABT-888 (A 0.125–0.5 μM), cells treated with 64 nM SN38 in combination with 0.5 μM VE-821 (S.5V) and varying concentrations of ABT-888 (0.125–0.5 μM) and cells treated with 64 nM SN38 in combination with 1 μM VE-821 (S1V) and varying concentrations of ABT-888 (0.125–0.5 μM). ^*^Significantly different from untreated control, ^γ^significantly different from cells treated with SN38 alone. *p* ≤ 0.05.

The 3 colon cancer cell lines were treated with SN38, ABT-888 and/or VE-821 alone or various combinations of the three drugs for 24 h and assayed for PARP enzymatic activity (Figure 4). 64nM SN38 significantly increased PARP activity in these cells. ABT-888 dramatically reduced PARP activity in the presence and absence of SN38 in a dose-dependent fashion. Treatment with VE-821 alone was also associated with increased PARP activity in HCT-116 and LoVo cell lines. In contrast, cells treated with SN38 combined with VE-821 had lower levels of PARP activity than cells treated with SN38 alone in HCT-116 cells and to a lesser extent in LoVo cells (*p* ≤ 0.05) but not in HT-29 cells. Moreover, HCT-116 cells treated with combinations of SN38/ABT-888/VE-821 had the lowest levels of PARP activity compared to cells treated with SN38 combined with ABT-888 (*p* ≤ 0.05) (Figure 4A), while this was not seen with the other two cell lines (Figures 4B,C).

### VE-821 and/or ABT-888 decreased SN38 induced Chk1 phosphorylation

Exposing HCT-116 cells to increasing concentrations of VE821 (0.25–16 μM) combined with 64nM SN38 caused a progressive reduction in Chk1 phosphorylation (Ser317) plateauing at 1 μM.

**Figure 5:**
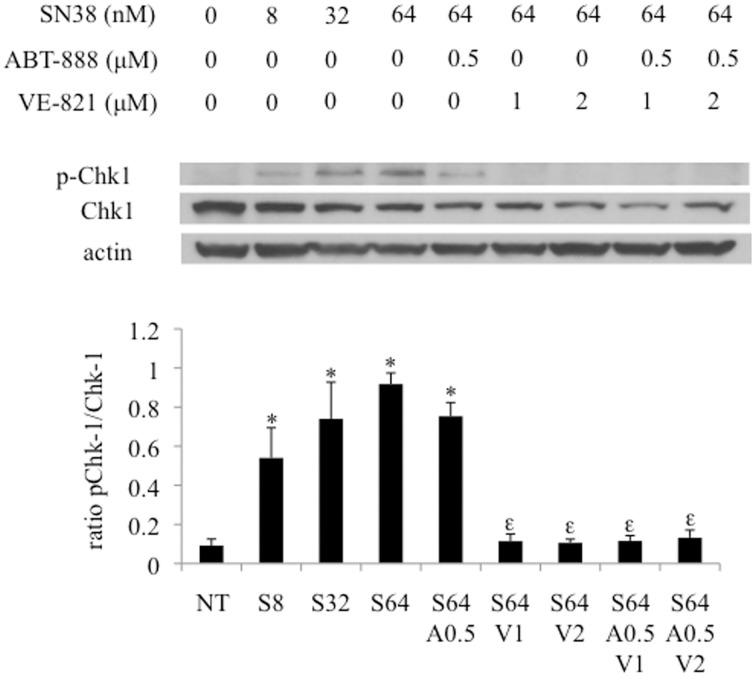
**Effect of combination drug treatments (24 h) on the expression and phosphorylation status of Chk-1 (S317) in HCT-116 cells as determined by western blot analysis, representative of 3 replicates**. NT = vehicle treated control, S8 = 8 nM SN38, S32 = 32 nM SN38, S64 = 64 nM SN38, A0.5 = 0.5 μM ABT-888, V1 = 1 μM VE-821, and V2 = 2 μM VE-821. ^*^Significantly different from NT cells, ^ε^significantly different from S64 cells, *p* ≤ 0.05.

The effect of the combination treatment of PARP and ATR inhibitors plus SN38 was assessed by western blotting samples in the HCT-116 cell line (Figure 5). While ABT-888 in combination with SN38 demonstrated a reduction in phosphorylation of Chk1, VE-821 at 1–2 μM largely suppressed SN38–induced Chk1 phosphorylation as previously described (Fokas et al., 2014) (Figure 5). The addition of ABT-888 had no further effect on VE-821 suppression of SN38-induced Chk1 phosphorylation (Figure 5).

### pDNA-PK is increased in response to ATR inhibition in a PARP dependent manner

**Figure 6:**
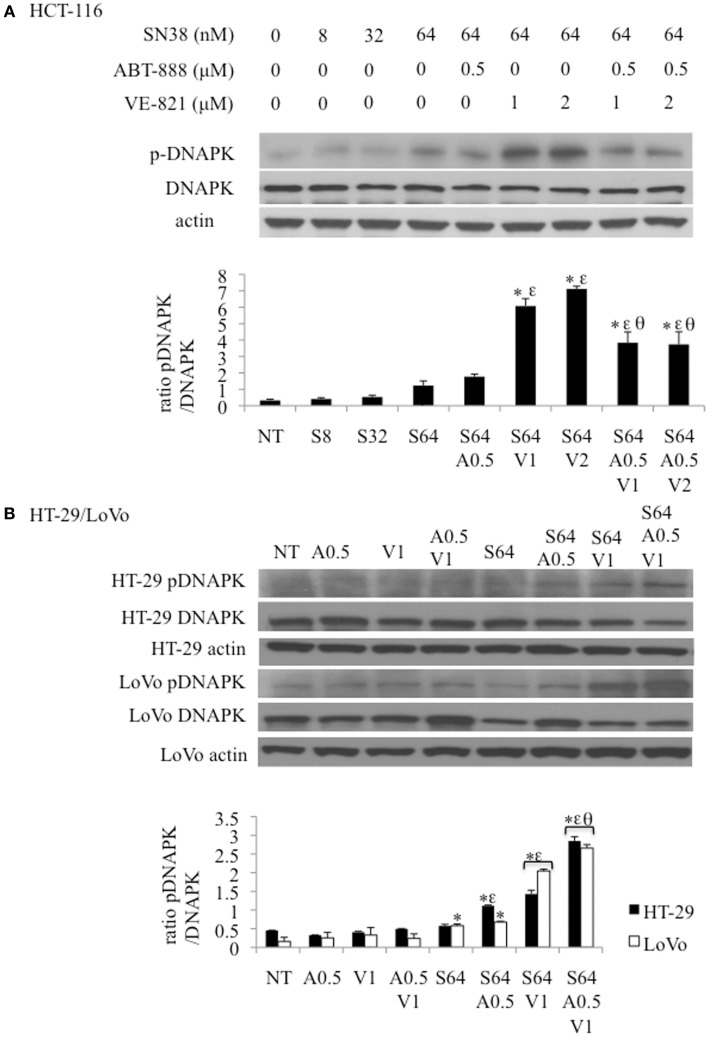
**Effect of combination drug treatments (24 h) on the expression and phosphorylation status of DNA-PK (S2056) in 3 colon cancer cell lines as determined by western blot analysis, representative of 3 replicates**. **(A)** HCT-116 cells treated with: NT = vehicle treated control, S8 = 8 nM SN38, S32 = 32 nM SN38, S64 = 64 nM SN38, A0.5 = .5 μM ABT-888, V1 = 1 μM VE-821, and V2 = 2 μM VE-821. **(B)** HT-29 (black bars) and LoVo (white bars) cells treated with S64 = 64 nM SN38, A0.5 = 0.5 μM ABT-888, V1 = 1 μM VE-821 alone or in combinations as indicated. ^*^Significantly different from NT cells, ^ε^significantly different from S64 cells, ^θ^significantly different from cells treated with SN38 and corresponding levels of VE-821, *p* ≤ 0.05.

In addition to p-Chk1, western blot analysis was also done for pRAD51 and p-DNA-PK to determine the effects of SN38, ABT-888, and VE-821 treatment on HRR and NHEJ, respectively (Figure 6). p-RAD51 protein levels increased with SN38 treatment in a dose dependent manner but did not change with addition of either ABT-888 or VE-821 (Supplemental Figure 1).

The levels of p-DNA-PK increased incrementally with the SN38 concentration. SN38 (64 nM) combined with 1 or 2 μM VE-821 caused a substantial increase in DNA-PK phosphorylation compared to SN38 alone in all 3 cell lines (Figures 6A,B) This response was diminished by the addition of 0.5 μM ABT-888 to the SN38/VE-821 combinations only in HCT-116 cells (Figures 6A,B).

## Discussion

Improvements in understanding of DNA repair pathways and their relationships has facilitated the development of several targeted therapies aimed at sensitizing neoplastic cells to the effect of DNA-damaging chemotherapy (Reaper et al., 2011). As such, the ATR-Chk1 pathway is a potential target for anti-cancer therapy and ATR-selective inhibitors are currently in development. These inhibitors hold significant potential because the ATR-Chk1 pathway plays important roles in regulating cell cycle arrest, NHEJ, and HRR (Fokas et al., 2014; Chen et al., 2012).

Our results revealed that a combination of both a PARP and an ATR inhibitor at non-toxic concentrations plus SN38 produced a dramatic 4.5–27-fold decrease in the IC_50_ of SN38 across the various cell lines with the largest decrease occurring in HCT-116 cells. The addition of each inhibitor separately to SN38 enhanced its cytotoxic effect, but to a lesser extent than when cells were treated with all three drugs. 0.5 μM ABT-888 was used in this work because in a previous study, higher concentrations (1–4 μM) did not provide additional sensitization to SN38 (Davidson et al., 2013). In agreement with the cytotoxicity results, G_2_-M arrest was most significant in cells treated with all three drugs.

In addition to cell cycle arrest, combination drug treatments also increased DNA damage (DSBs) as indicated by increased levels of γH2AX, which is an indicator of DNA double strand breaks (Paull et al., 2000). The highest levels of γH2AX were observed in response to combination treatment with all three drugs, most likely due to decreased DNA repair capacity in the presence of the inhibitors.

VE-821 stimulated PARP activity in the HCT-116 and LoVo cell lines is possibly secondary to unresolved replication stress due to the absence of ATR activity. This was not observed in the HT-29 cells and may be related the fact that they are p53 mutated with altered replication stress signaling. As expected the PARP inhibitor ABT-888 decreased PARP activity both in the absence and presence of SN38. The two inhibitors together decreased maximally the PARP activity only in the HCT-116 cells. This may be secondary to decreased ATM levels in HCT-116 cells since depletion of ATM in breast cancer cells confers sensitivity to PARP inhibition (Gilardini et al., 2013).

Interestingly, SN38 induced autophosphorylation of DNA-PK was increased in all three cell lines in the presence of the ATR inhibitor VE-821. The increase in pDNA-PK is likely a mechanism to compensate for the lack of ATR activity at stalled replication forks in the presence of SN38. We speculate that reduced ATR activity in the presence of the ATR inhibitor prevented repair of DNA damage resulting from SN38 activity leading to the formation of DSBs and increased recruitment and autophosphorylation of DNA-PK. This autophosphorylation of DNA-PK was diminished when ABT-888 was used in combination with SN38 and VE-821 only in the HCT-116 cells. Interaction of DNA-PK and PARP has been shown to cause substantial conformational changes in the DNA-PK synaptic dimer (Spagnolo et al., 2012). Furthermore, DNA-PK is ADP-ribosylated by PARP which stimulates the activity of DNA-PK (Ruscetti et al., 1998). HCT-116 cells have decreased levels of ATM as compared to the two other cell lines (Kim et al., 2002; Zhou et al., 2014). Also in the presence of an ATM inhibitor induction of DSBs in human cells decreased autophosphorylation of DNA-PK (Zhou and Paull, 2013). This suggests that the decrease in phospho-DNA-PK seen in the presence of two inhibitors together with SN38 only in the HCT-116 cells may be related to the decreased levels of ATM in these cells.

As such, the observed sensitization effect of both inhibitors utilized with SN38 in the colon cancer cell lines may be related to the combined inhibition of both pathways. Since PARP-1 and ATR co-immunoprecipitated in extracts prepared from MMS-treated cells implying a potential direct interaction between these pathways (Kedar et al., 2008) this interaction may be altered in the presence of these inhibitors in the presence of SN-38 such that there is more sensitization. This effect is optimal in the HCT-116 cells as a result of both optimal suppression of PARP activity and attenuation of DNA-PK activation possibly due to decreased ATM levels. Interestingly, a recent clinical study associated low ATM expression in colon cancer with poor clinical outcomes (Beggs et al., 2012). Our data suggests that the combination of ABT-888, VE-821, and SN38 may improve chemotherapy in these patients.

## Conclusion

Chemotherapy remains the mainstay of cancer treatment. Treatment strategies to enhance cytotoxicity or minimize adverse events have emerged and are being developed. Among small molecule inhibitors used to improve the therapeutic index, ABT-888, a PARP inhibitor, and VE-821, a selective ATR inhibitor, were examined. Combination treatment with these inhibitors at nontoxic concentrations with SN38 resulted in the greatest chemosensitization along with increased DNA damage. The present data suggest that this combination may effectively sensitize some colon cancers to irinotecan-based chemotherapy.

## Funding

This research was supported in part by a Collaborative Health Research Project grant (#2970) from the Natural Sciences and Engineering Research Council of Canada. We also gratefully acknowledge support from private donations provided by: Mr. Michael Perelshtein and Michael and Pat Heller of Beamish House.

### Conflict of interest statement

The authors declare that the research was conducted in the absence of any commercial or financial relationships that could be construed as a potential conflict of interest.
