# Supplementary material for: Simultaneous inhibition of ATR and PARP sensitizes colon cancer cell lines to irinotecan
Source: Front Pharmacol. 2015 Jul 22;6:147. doi: 10.3389/fphar.2015.00147 (PMC4510998; doi:10.3389/fphar.2015.00147)
Supplement: Supplementary file 1 [file Table1.DOCX]

Supplementary Table 1

| Cell Line | VE-821  (µM) | ABT-888  (µM) | SN38 (IC_50_) ± SE  (nM) | R value | P value |
| --- | --- | --- | --- | --- | --- |
| LoVo |  |  | 13.5±1.2 |  |  |
|  | 1 |  | 4.5±1.9 | 3.0 | 0.02 |
|  | 0.5 |  | 5.8±2.0 | 2.3 | 0.03 |
|  |  | 0.5 | 9.4±1.0 | 1.4 | 0.06 |
|  | 1 | 0.5 | 3.0±1.2 | 4.5 | 0.004 |
|  | 0.5 | 0.5 | 4.7±1.9 | 2.9 | 0.02 |
|  |  |  |  |  |  |
| HCT-116 |  |  | 8.1±0.7 |  |  |
|  | 1 |  | 3.5±0.5 | 2.3 | 0.006 |
|  | 0.5 |  | 5.2±0.6 | 1.6 | 0.03 |
|  |  | 0.5 | 3.9±1.1 | 2.1 | 0.03 |
|  | 1 | 0.5 | 0.3±0.03 | 27 | 0.0004 |
|  | 0.5 | 0.5 | 2.1±0.4 | 3.9 | 0.002 |
|  |  |  |  |  |  |
| HT-29 |  |  | 20.5±1.8 |  |  |
|  | 1 |  | 9.3±0.6 | 2.2 | 0.004 |
|  | 0.5 |  | 12.2±1.3 | 1.7 | 0.02 |
|  |  | 0.5 | 12.9±0.7 | 1.6 | 0.02 |
|  | 1 | 0.5 | 3.9±0.5 | 5.3 | 0.0009 |
|  | 0.5 | 0.5 | 6.1±0.2 | 3.4 | 0.001 |

Supplementary Table 1 Colon cancer cell lines LoVo, HCT-116 and HT-29 were treated with SN38 alone or in combination with various concentrations of the ATR inhibitor VE-821 and/or the PARP inhibitor ABT-888. The IC_50_ values of SN38 were significantly reduced in the presence of VE-821 and/or ABT-888 for all cell lines. Drug sensitization (R) values were determined, according to Willmore (Willmore et al., 2008), by calculating the ratio: (IC_50_ SN38 alone/IC_50_ SN38 with inhibitors); R>1 = sensitization.

Supplementary Table 2

| Cell line | ABT-888 IC_50_ (µM)±SE | VE-821 IC_50_ (µM)±SE |
| --- | --- | --- |
| HCT-116 | 38±0.8 | 10.5±1.3 |
| HT-29 | ≥100 | ≥100 |
| LoVo | 49.53±5.5 | 60.5±29.5 |

Supplementary Table 2 The IC_50_ values of ABT-888 and VE-821 alone in different colon cancer cell lines.
