# Supplementary material for: Simultaneous inhibition of ATR and PARP sensitizes colon cancer cell lines to irinotecan
Source: Front Pharmacol. 2015 Jul 22;6:147. doi: 10.3389/fphar.2015.00147 (PMC4510998; doi:10.3389/fphar.2015.00147)
Supplement: Supplementary file 2 [file Image1.PDF]

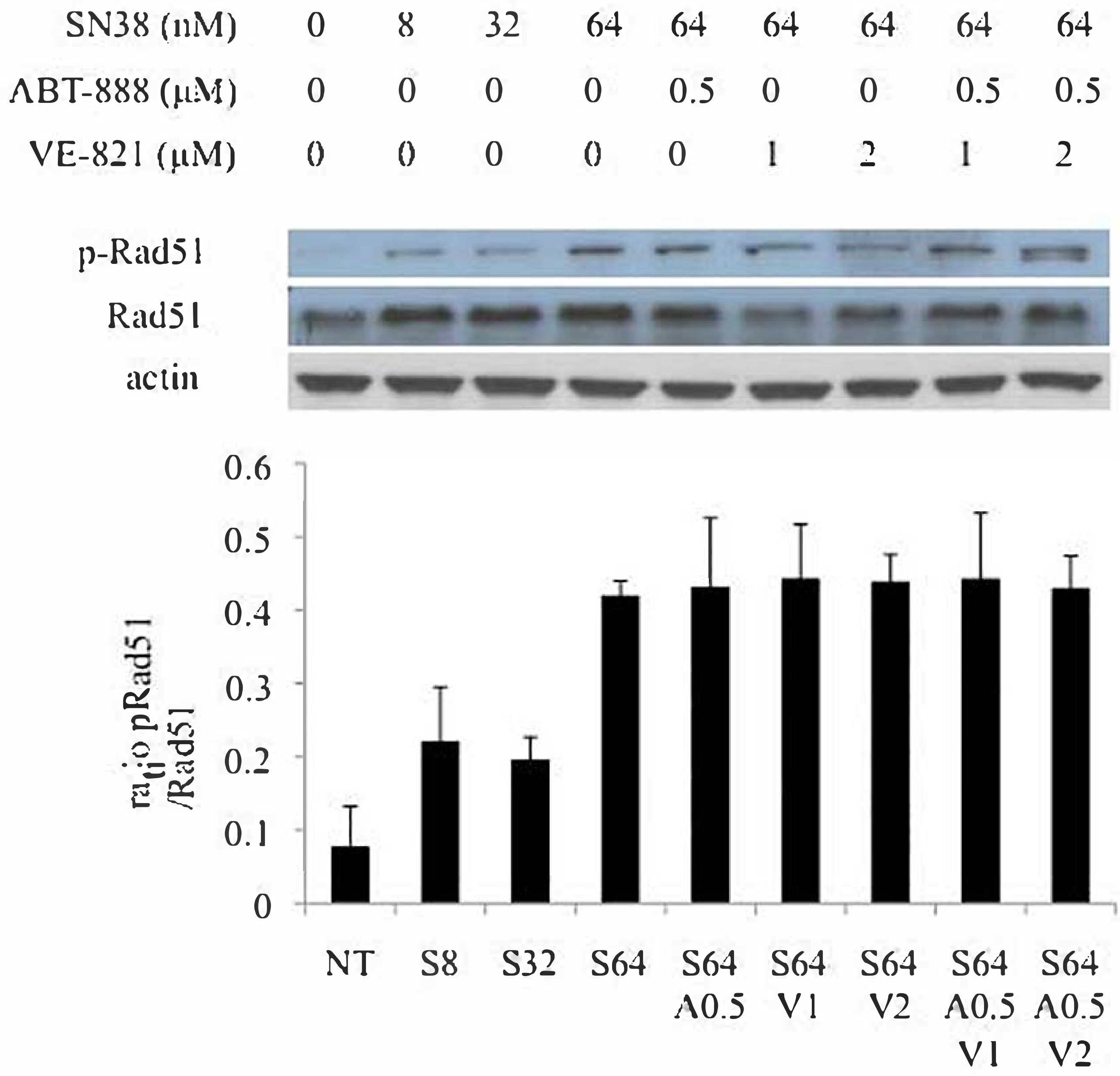

Supplemental Figure 1 Effect of combination drug treatments (24h) on the expression and phosphorylation status of Rad5 1 (T 309) in HCT-116 cells as determined by westernanalysis, representative of 3 replicates. NT = vehicle treated control, S8 = 8nM SN38, S32 = 32nM SN38, S64 = 64nM SN38, A0.5 = 0.5μM ABT-888, V1 = 1 μM VE-821 and V2 = 2μM VE-821. * = significantly different from NT cells, p≤0.05.
